# Implementation and evaluation of participatory advisory boards in mental health research: a research protocol of the ‘PART-Beirat’ project

**DOI:** 10.1186/s40900-023-00522-6

**Published:** 2023-12-06

**Authors:** Fenia Ferra, Eva Drewelow, Olga Klein, Marcel Daum, Peggy Walde, Kai Gerullis, Ingo Kilimann, Jack Tomlin, Stefan Teipel, Birgit Völlm

**Affiliations:** 1 Clinic of Forensic Psychiatry, Rostock University Medical Centre, Rostock, Germany; 2 Deutsches Zentrum Für Neurodegenerative Erkrankungen (DZNE) Rostock/Greifswald, Rostock, Germany; 3 Department of Psychosomatic Medicine, Rostock University Medical Center, Rostock, Germany; 4 School of Law and Criminology, University of Greenwich, London, UK

**Keywords:** Participatory research, Patient and public involvement (PPI), People with lived experience (PWLE), Advisory boards, Dementia, Forensic mental health, Secure hospitals

## Abstract

**Background:**

The use of participatory research approaches in the field of dementia and forensic mental health research has been on the rise. Advisory board structures, involving people with lived experience (PWLE), have frequently been used for guiding and leading research. Yet, there has been limited guidance on the establishment, retention and use of advisory boards in the field of dementia and forensic mental health research.

**Objective:**

This project outlined in this research protocol will investigate the benefits and challenges of establishing three patient advisory boards, involving PWLE, practitioners and researchers with the purpose to guide research. Data will be used to develop guidelines for best practice in involving PWLE in dementia and forensic mental health research through advisory boards.

**Methods:**

The research project will be divided into three phases: Phase I will involve two topic-specific systematic reviews on the use of participatory research with PWLE, followed by an initial study exploring PWLE’s, practitioners’ and researchers’ expectations on research involvement. Phase II will consist of the establishment of three advisory boards, one focusing on dementia, one on forensic mental health and one overarching coordinating advisory board, which will involve PWLE from both fields. Phase III, will consist of interviews and focus groups with advisory board members, exploring any challenges and benefits of involving PWLE and practitioners in advisory boards for guiding research. To capture the impact of involving PWLE in different research phases and tasks, interviews and focus groups will be conducted at four different points of time (0, 6, 12, 18 months). Reflexive thematic analysis will be used for the analysis of data.

**Discussion:**

The project aims to explore the involvement of PWLE and practitioners in guiding research and aims to develop guidelines for best practice in establishing and using patient advisory boards in dementia and forensic mental health research and involving PWLE and practitioners in research.

## Abstract (plain language summary)

There is an increasing involvement of people with mental health issues in research, especially in the form of advisory boards. So far People With Lived Experience [PWLE] of mental health issues acquired either from first person experience or through family members, and mental health practitioners’ involvement in research has been found to benefit research and society. This is because it increases reach and quality of research, whilst raising the voice of people commonly excluded from decision making (e.g. research, care provision). This research protocol describes the design of a three-year research project. The project aims to establish and use patient advisory boards, involving PWLE, practitioners and researchers, to guide research. The project will consist of three phases: (1) a review of previous studies on the use of participatory research with PWLE of dementia and forensic mental health care, followed by an initial study exploring PWLE’s, practitioners’ and researchers’ expectations in research involvement, (2) the establishment of three advisory boards, one focusing on dementia, one on forensic mental health and one overarching coordinating advisory board, and (3) an exploration of challenges, barriers and benefits of involving PWLE of dementia/forensic mental health care and practitioners in advisory boards for research through interviews and focus groups. Interviews with PWLE and practitioners involved in the advisory boards will be conducted at four different points of time (0, 6, 12, 18 month). At the end, we aim to develop guidelines for establishing advisory board structures, involving PWLE and practitioners in research.

## Background

Involving People With Lived Experience (PWLE)^1^ in research comes with several benefits and challenges, for both research and the wider community [24, 31]. Evidence shows that involving PWLE in research increases recruitment rates of participants and assists researchers in obtaining funding, developing study protocols, and selecting appropriate outcome measures [14], while others have suggested that the exclusion of people affected by the topics under investigation in research reduces the democratic legitimacy of publicly funded research and raises questions about the utility of scientific knowledge (NIHR [37].

From its early days and throughout their long history, participatory research approaches have been used with seldom-heard and marginalised groups, as “co-producing research with marginalised populations has value in addressing gaps in knowledge that arise from exclusion” [40]. Some examples include research with individuals living with disabilities or special needs [11], children with disabilities [56], sexual and gender-based violence [58], asylum seekers and conflict affected populations [43]. In recent years, there has been an increase of participatory research used with people living with dementia and their caregivers (e.g. [25, 36, 41], and within forensic settings, prisons and forensic psychiatric hospitals (e.g. [18, 35]. This is particularly interesting as both people living with dementia and people having spent time in forensic settings or hospitals have suggested to commonly face multiple marginalisation and social exclusion [7, 50]. As such, we believe these two groups might constitute a good example on exploring how participatory research methods can effectively be applied in mental health research.

Dementia affects 55 million people worldwide [59], and as such, it constitutes a priority in the field of mental health research. Participatory research approaches have been used for the identification of research priorities [6, 25, 28, 30], analysis of data involving informal caregivers of people living with dementia [29], analysis of communication and collaboration in dementia care [44], and development of assistive technologies [26, 38]. Research has stressed the benefits of involving PWLE in research, both for PWLE, on an individual level, and knowledge production (e.g. [19, 28, 30]. Despite the increase on the use of participatory research approaches, there is still limited evidence on what works best for involving PWLE of dementia in research [13].

Similarly, participatory research approaches have been effectively used in forensic settings, prisons [10, 23] and forensic psychiatric hospitals [35] in different countries, including the UK [20], Ireland [47], New Zealand [55] and Canada [16]. Studies have identified several practical and ethical barriers (e.g. [22]), with most associated to the restrictive and authoritarian nature of forensic settings [49]. A rapid review [51], which explored how forensic mental health patients have been involved in research, stressed that there is a need for more research on how to effectively and meaningfully involve PWLE of forensic mental health care in research.

There have been several examples of how PWLE and practitioners^2^ have been involved in mental health research. PWLE have sometimes been involved in specific research stages (e.g. [27]) or have been engaged throughout the whole research process, including research design (e.g. [3, 32]. Advisory boards and committees have constituted an effective way to involve PWLE and practitioners in mental health research (e.g. [39, 46], and more particularly in the field of dementia [21, 57] and forensic mental health [3]. Yet, there is limited guidance on the process of establishing, retaining, and using advisory board structures (an example coming from HIV prevention and treatment research, [54]. This is particularly important for Germany, where there is limited previous research on establishing research advisory boards in dementia and forensic mental health. Advisory boards provide the opportunity for PWLE and practitioners to be actively involved in all stages of the research process and give space for communication,while more stable advisory board structures might also ensure continuity (in research agenda and goals and promote social change by increasing PWLE’s participation in research [42].

### Aims

The project outlined in this protocol aims to establish and evaluate two topic-specific advisory boards, one focusing on dementia and one on forensic mental health, as well as an additional overarching coordinating advisory board; these will all form the “PART-Beirat”.^3^ The advisory boards will involve PWLE, researchers and practitioners in the fields of dementia and forensic mental health. The advisory boards will be used for the design, implementation and dissemination of research projects in the two topics of interest. We aim to evaluate the establishment and use of advisory boards by exploring participants’ perspectives on their involvement on the advisory boards and in guiding research. At the end of the project, we aim to develop guidelines for best practice on establishing topic-specific advisory boards, with the purpose of involving PWLE and practitioners in research guidance, design and implementation.

## Methods

### Context

The study involves work in two different topics of mental health, and in two different departments in a University Hospital.

1. Dementia

The Section of Geriatric Psychosomatics and Dementia offers a memory clinic at Rostock University Medical Center. Patients with memory concerns undergo guideline-based diagnostics and receive treatment and care with a referral from their general practitioner or specialist. Patients are also informed about current research studies. The memory clinic sees up to 400 patients per year.

2. Forensic mental health

The Hospital for Forensic Psychiatry at Rostock University Medical Center consists of 7 wards and has a 103 bed capacity. The hospital accommodates male and female individuals with a range of mental health diagnoses, including substance use disorders, who have committed offences in the context of their mental disorder. Upon release, patients are followed by an aftercare unit.

### The research team

The research team involves four researchers working in the field of dementia, with a background in psychiatry, psychology, neurology, five researchers in forensic mental health, with a background in psychiatry, psychology, criminology and sociology and one PWLE in forensic mental health care, currently working as a peer support worker at the Hospital for Forensic Psychiatry, Rostock University Medical Center. All members of the research group are white, five members of the research team are female and five are male.

### Phases of the project

**Fig. 1:**
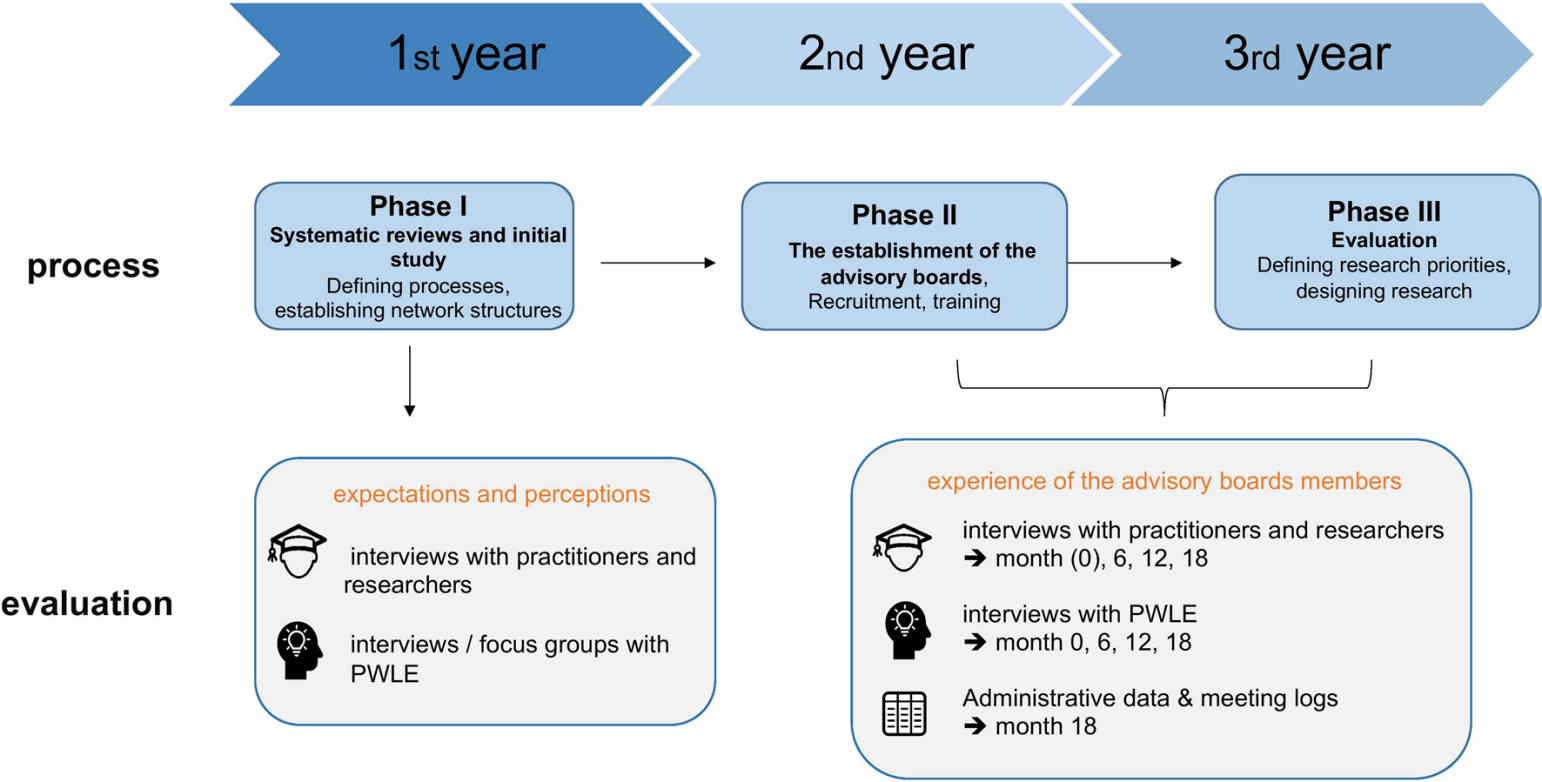
The “PART-Beirat”, How data will be collected

The project will run for approximately three years and will be divided into three phases: Phase I, two systematic reviews and an initial study, Phase II the establishment of the advisory boards, Phase III evaluation (Fig. 1).

#### Phase I

Phase I will be divided into two stages: stage 1, two topic-specific systematic reviews and stage 2, an initial explorative study. Two systematic reviews will be conducted exploring how participatory research has been utilized with a) PWLE in forensic mental health care and b) PWLE in dementia; both registered in PROSPERO. The systematic reviews will inform the later stages of the project. The second stage of this phase will involve a study consisting of interviews and focus groups with PWLE, as well as interviews with practitioners and researchers. This, together with available guides and standards on public involvement on research (e.g. [53]) and research boards [15] will guide the development of the advisory groups (and related practices) and later the identification and recruitment of potential participants for advisory boards through existing networks (e.g. self-help groups, well established professionals in the field). We aim that the advisory boards, when formed, will identify research priorities and proceed to the design of relevant research.

### Sample

A purposive sampling will be employed. We aim to recruit 10 practitioners/researchers and about 20 PWLE at this stage of the project. We will invite established practitioners, and researchers to participate in the study. Academics and practitioners with expertise in the fields of dementia and forensic mental health in Germany will be invited to participate. PWLE will be (a) resident in the Hospital for Forensic Psychiatry at Rostock University Medical Center or recently discharged (followed by the after care clinic), or (b) recruited via the Memory Clinic of Rostock University Medical Center. Prospective participants will be identified by clinical staff (holding a dementia or cognitive impairment diagnosis from the memory clinic will be a prerequisite). Potential participants will be briefly informed about the study. if they express an interest to participate, an individual consultation with a member of the research team will be arranged, so the prospective participant can be provided with all relevant information and have the opportunity to ask any questions they might have. We will follow Bates and Ward’s [4] guide on informed consent. All participants will be asked to provide written and verbal (at the beginning of the interview/focus group) consent. Individuals found unable to give informed consent by medical staff will not be included in the study. Only family members and/or carers of people living with dementia will be recruited as PWLE, no family members and/or carers of PWLE of forensic mental health care will be involved in this study. As PWLE of forensic mental health care families’ perspectives (as well as their involvement in research) is an understudied and complex topic [45], and considering the project’s timeline and available resources, we thought that focusing on both PWLE of forensic mental health care and their families at the same time would not be feasible. As potential participants will be informed about the study and invited to participate on individual consultations, no adverse impact is expected in case they refuse to participate. Researchers will provide thorough explanation of the voluntarily nature of participation. Moreover, researchers will explicitly state that involvement on the study will have no impact on their care and/or relationships with therapeutic personnel.

### Procedure

The initial study will involve a number of semi-structured interviews (n ≈ 15, estimated) with PWLE, practitioners, researchers and two focus groups (n ≈ 2 estimated, involving 6–8 participants each) with PWLE. The interviews will be conducted by members of the research team. Different types of data collection with PWLE will be employed to better accommodate individuals’ needs. More particularly, focus groups will be conducted with PWLE in forensic mental health care and interviews with PWLE in dementia. While we thought that focus groups might be a better way to capture individuals’ perspectives, as they promote an active dialogue and sharing between participants, they might not be suitable for people living with dementia. This is because we expect that there will be a variation of health conditions and needs, which we might fail to effectively cover in a group context, focus groups. We wanted to ensure that all participants will have equal opportunities to freely share their thoughts, but due to their health conditions, some individuals might face increased difficulties in communicating their thoughts in a group setting. Moreover, as people living with dementia might be accompanied by carers, we thought that this would also constitute individual interviews with this group of participants a better choice at this stage. The number of interviews with people living with dementia and their carers might vary, as interviews will be either conducted on an individual or dyad, patient/carer, basis.

PWLE in dementia will be interviewed at the Clinic at a convenient time for them. In case of cognitive impairments or other reasons that do not allow interviewing patient(s) (e.g. personal request) on an individual basis, PWLE might be interviewed together with their carers/family members. This is to ensure PWLE’s safety and protect their wellbeing. Tandem-interviews are a good way to protect PWLE from being overwhelmed and to support them before, during and after the interview.

PWLE in forensic mental health care will involve focus groups, which will be co-facilitated by a researcher and a peer support worker currently working at the Forensic Clinic, both core members of the project’s team. Practitioners and researchers will be interviewed in one of the Clinics at a convenient time for them or by telephone. Both interviews and focus groups will be audio-recorded and participants’ permission will be requested before and during the start of the interviewing process. In the event of no permission to audio recording on individual interviews, note taking will take place instead. As note taking in focus groups might reduce accuracy and credibility of data collected, due to the amount of people and information involved, participants that will not consent on audio-recording will be excluded from the process and provided the opportunity to be individually interviewed. All participants will be provided with a debrief sheet, containing information about the project as well as contact details of members of the research team. At the end of interviews/focus groups all participants will have the chance to ask any questions they might have (e.g. about the project, data retention). All interviews and focus groups will be transcribed verbatim and anonymised. Data will be stored in a password-protected folder on the university premises and only the project team will have access.

Both interviews and focus groups with PWLE, practitioners and researchers will cover the following themes, and are expected to range between 45 and 120 min:

1. Knowledge and prior experience of being involved in research
2. Benefits/challenges of using participatory research approaches (including advisory boards) in dementia/forensic mental health
3. Benefits/challenges/barriers for PWLE/practitioners/researchers, when PWLE/practitioners are involved in research
4. Any context related barriers

#### Phase II

Phase II will involve the establishment of the two topic-specific advisory boards, and one overarching one, bringing people from the two different topics together. Participation of individuals from different social groups (e.g. age, gender, socio-economic, religious, ethnic) will be promoted.

### Sample

People involved in the previous phase of the project (Phase I) will be invited to take part. Other practitioners, such as physicians, psychologists, peer support workers, and academics will also be invited. Prospective participants will need to be based in North Germany, to ensure ease of participation and avoid later withdrawals (e.g. commuting for face-to-face interviews and meetings). Snowball sampling will also be employed for the recruitment of participants for the advisory boards**.** Experience in the field of patient participation and/or participatory research will not be a prerequisite for participation. Each topic-specific advisory board will involve up to 10 members, who will be practitioners, researchers and PWLE. In the dementia advisory board, carers of PWLE will also be involved. Participants will all be adults, and there will be no other age restrictions. The size of these topic-specific boards will be flexible and may vary. We aim, however, to keep a good balance (equal split) between the number of researchers, practitioners and PWLE being involved in each advisory board.

The recruitment of PWLE will be carried out by researchers from the research project. Similar to Phase I, prospective participants will be identified by clinical staff. Suitable candidates from the field of dementia will be recruited through direct contact in the memory clinic and from, working within the Clinic for Psychosomatic Medicine and Psychotherapy (KPM) at Rostock University Medical Center. Suitable candidates from the field of forensic mental health will be approached directly in the Clinic for Forensic Psychiatry at Rostock University Medical Center or in the outpatient department by the research team.

While we intent to keep the advisory boards stable, in terms of the number and people involved, in case there is a drop-out or in case of any other unforeseen circumstances that will result in a drop-out, we will aim to recruit new members. Any relevant decisions, as well as the recruitment process will be led by the advisory boards.

### Procedure

Members will be convened to meet regularly in pre-set face to face meetings. During the initial meetings, ground rules will be established and training on relevant aspects of research involvement will be provided to all members. We aim to provide trainings at the beginning of the project on advisory groups, operation and purpose, as well as research methods. There will be further trainings provided throughout the whole project. Training will be based on previous work (e.g. [33, 48] and designed and delivered by members of the research team and overarching advisory board. All content will be provided in a way, which is suitable for PWLE of dementia and forensic mental health care (e.g. information provided on a plain language, avoiding jargon, using different forms of presentation). The two topic-specific advisory boards will identify research priorities and develop their own research agenda. A designated member with lived experience (different in each group) will chair these meetings. There will be an opportunity to involve co-chairs, if needed.

Some of the main tasks of topic-specific advisory boards will include:

- Identifying research priorities
- Input into designing research projects
- Advise on recruitment approaches
- Design of recruitment and other research relevant materials (e.g. questionnaires)
- Target group-specific dissemination of research projects’ results

In addition to the two topic-specific advisory groups, a coordinating overarching advisory board will also be established. The coordinating advisory board will comprise of 9–12 members and will be chaired by an ‘involvement lead’ (the project manager of the project). It will include the chairs (and co-chairs if any) of the topic-specific advisory boards, other PWLE, practitioners and researchers from both advisory boards, as well as researchers working on this project.

The main goal of the coordinating advisory board will be the continuous monitoring and support on all activities of the topic-specific advisory boards. It will provide the opportunity to bring people from different fields of mental health together. More precisely, we aim that the coordinating advisory board will build a bridge between the topic-specific advisory boards, research and clinical institutes involved, organisations and community. In addition, this group will lead on training design and provision.

Some of the main tasks of the coordinating advisory board will include:

- supporting and training PWLE and researchers in the participation process
- promoting the involvement of PWLE in research
- developing and maintaining a network of academic institutions, patient advocacy groups and charitable organisations
- carrying out administrative tasks
- public relations work (e.g. preparing reports, press releases)

We aim to provide (and promote) flexibility and we will be willing to make any relevant arrangements for the operation of the advisory boards. Members of the advisory boards will decide on the time they will be able to spend on the boards, and the tasks they would like to get involved (there will be a minimum time needed set during the establishment of the advisory boards).

#### Phase III

In Phase III it is anticipated that the advisory boards will already be working on identifying research priorities, and designing and guiding research. Advisory boards will decide how they will operate and work as groups. Duration and frequency of meetings might vary and will be based on advisory boards members’ needs. All projects will be monitored by the coordinating advisory board.Information about priorities identified, and designing and guiding research and the designing process will be collected. Results coming from previous phases of the project will be used to inform practices in this phase, as well as the development of guidelines for best practice in involving PWLE and practitioners in research.

### Procedure

After the establishment of the advisory boards, there will be set time points (0, 6, 12, 18 month)^4^ when data will be collected. The first data collection point will be after the establishment of the advisory boards (Month 0, Phase I), the second after the initial meetings (Month 6), the third one after 6 months (Month 12) and the last one towards the end of the project (Month 18) (see Fig. 1*)*. Data will be collected through semi-structured interviews, and will focus on:

1. Exploring participants’ experiences and perceptions of being part on the advisory boards (organizational structure, inclusivity, group dynamics, developing research ideas)
2. Identifying benefits/challenges and barriers in guiding research

The interviews will be conducted either in the Hospital for Forensic Psychiatry or the Memory Clinic at Rostock University Medical Center and will follow the same procedure as in initial study (informed consent, recording, debrief). The duration of the individual interviews is expected to range between 30 and 60 min, but may be shorter for PWLE of dementia, depending on their medical and mental health needs.

In addition to interviews, an analysis of key administrative data (meeting schedules, logs etc.) will also be performed and presented. Information on the work of advisory boards, including meetings (number of participants, content, duration) and information on projects initiated by the advisory boards, as well as any operational changes made as a result of the work of the boards will be included in the analysis.

### Ethical issues and data protection

The study has received an ethical approval by the ethics committee of Rostock University Medical Center (A 2023–0007).

Prior to the date of interviews and focus groups, and upon their agreement to participate, prospective participants will be provided with a participant information sheet. They will be allowed sufficient time to go through it and raise any questions and/or clarifications, they might have. At the beginning of interviews/focus groups prospective participants will again be verbally briefed about the study (e.g. aims and objectives, what participation will entail) and they will be informed about their right to withdraw.

Ensuring anonymity of participants throughout the project will be a priority. In both interviews and focus groups, transcripts will be anonymised. In focus groups, prospective participants will be informed about their right to keep their anonymity and will be provided the opportunity to use a pseudonym during their involvement in the advisory boards and/or project.

Participants involved in the two topic-specific advisory boards, and the coordinating advisory board will be reimbursed for the time they will give on the project.

#### Analysis

All data will be analysed inductively on a latent level using reflexive thematic analysis [8, 9]. We will follow the 6 steps as outlined by Braun and Clarke [8]: 1.Familiarisation with the dataset, 2. Coding, 3. Generating initial themes, 4. Developing and reviewing themes, 5. Refining, defining and naming themes, 6. Writing up. The analysis will be underpinned by critical realism assuming that it is possible to arrive at reasoned judgments about truth and reality through rigorous research methods [2, 5]. The software MAXQDA for qualitative analysis of data will be utilized in our analysis. Analysis will be conducted by two members of the research group. Codes and themes will later be reviewed by other members of the research team and members of advisory boards to ensure validity.

All data collected will be used for the development of topic-specific guidelines for best practice in involving PWLE (a) dementia and their carers and (b) forensic mental health care in research advisory boards.

## Discussion

### Expectations & practical implications

Following on previous research conducted in other countries [1, 3, 57], it is anticipated that this project might have implications for research and mental health practice in Germany. Scholarly work has identified several benefits on involving PWLE in research, such as increased relevancy, insight, and on an individual level for PWLE building of self-esteem and skills (e.g. [22, 31]. This project outlined in this protocol aims to zoom in and explore the benefits of involving PWLE of dementia and forensic mental health care in research; two groups, which commonly face multiple marginalisation.

Involving PWLE in research has been thought to be a demanding and challenging task for both researchers and the community. For example, participatory research is a time-consuming process, which requires increased flexibility from all different sides. This is something that might come in conflict with common research timelines, and as such, have a negative impact on funding applications (e.g. [17]). At the same time, it might be too demanding in terms of time and effort for PWLE too. Moreover, dementia and forensic mental health scholars have talked about context specific challenges, such as the difficulty in doing research within forensic institutions, due to limited flexibility and power to operate within those [34]. We aim to try overcome those challenges, and get a more thorough look into those.

We hope that this project outlined in this protocol will help us get a better understanding of what works best in involving PWLE and practitioners in research and how to overcome relevant challenges. We hope that we will use the findings of this project to develop guidelines for establishing and utilising advisory boards, consisting of PWLE, practitioners and researchers, in the respected topics (dementia, forensic mental health care) in Germany.

### Limitations

Due to the study’s focus and design, requiring the involvement of field-experts and PWLE, the study will follow a purposive sample in all different phases, and as such, we do acknowledge that recruitment bias might occur. This is even more relevant considering the small number of people that we aim to involve, due to the qualitative and complex nature of the project.

Practitioners will consist of physicians, psychiatrists and psychologists, while other professionals working closely to PWLE of dementia/forensic mental health care, such as nurses will not be involved. This might be another limitation on the study’s findings and on the different perspectives the study will explore. Similar to that, PWLE will be selected based on health condition, and people with any severe symptoms, either dementia or any other mental health issues, will be excluded from the study. Considering the population of these two groups (high comorbidity for PWLE of forensic mental health care, and that dementia is progressive a disease), we do believe that this might again act as a limitation on the different perspectives we will access. However, as this is a novel project, we wanted to ensure efficiency and limit any potential barriers on individuals’ active involvement.

Moreover, even though interviews’ and focus groups’ rationales and guides will be discussed and decided among the research group, and we will aim on consistency, we do acknowledge that as interviews and focus groups will be conducted by more than one individual, we will not manage to limit the interviewer’s impact. As the project outlined in this protocol will focus on two different groups of people, PWLE of dementia and forensic mental health care, we thought that people with expertise in the respective area should lead data collection. Moreover, when possible (e.g. focus groups with PWLE of forensic mental health care), we aim to involve our research members with relevant living experience in data collection.

Even though we aim to promote diversity, and efforts will be made to ensure representation from different social groups (e.g. age, gender, ethnic background, religion), considering the regional nature of the project and the population we aim to involve, certain social groups might be under-represented (e.g. ethnic minorities). As an illustration, only 5% of the population of Hospital for Forensic Psychiatry at Rostock University Medical Center is coming from a different ethnic background than German. As such, it might be difficult to recruit people with diverse ethnic and/or cultural background. The study outlined in this protocol has a regional focus, North of Germany, and therefore, results of the project might have limited applicability in other socio-cultural and legal contexts. It might be important, for example, to consider how different care systems might affect relevant research in other parts of the world.

## Conclusion

Establishing the “PART-Beirat” in the North of Germany and developing its organisational structures, materials, and processes might provide a great insight into the involvement of PWLE and practitioners in research. This will be particularly relevant considering the recent rise of the use of participatory research in the topics under focus and the challenges of using participatory approaches effectively [12]. It will be the first study of this nature to be conducted in the North of Germany, and only one of very few in the whole country. As most studies on involving PWLE of dementia and forensic mental health care come from the UK and Canada, it might be interesting to see how involving PWLE in research can work in other places.

As noted earlier, due to the limited number of individuals involved in this project and its regional nature, certain sub-groups might be overlooked, and as such, future research should focus more on widening participation and ensuring that specific groups are equally represented and involved (e.g. other professionals, such as nurses, PWLE of diverse ethnic and cultural backgrounds). However, as there is currently limited knowledge on collaborate working with PWLE of dementia and forensic mental health care we do believe that the findings of this study might be of both interest and some applicability not only to Germany, but also to different socio-cultural contexts.

The project aims to develop guidelines for best practice for establishing and utilising advisory boards for guiding research, in the fields of dementia and forensic mental health, which is something that might be useful for future research in the region, and elsewhere. We do believe that it will be very interesting to see more research on involving PWLE in different socio-cultural and legal contexts in the future, as this is something that might enrich our understanding of effective and meaningful public involvement in research.

## Data Availability

Not applicable.

## Footnotes

1 PWLE is a term that has been used increasingly to describe people who have personal experience of the mental health issue under investigation, in participatory research. We acknowledge the use of this term poses risks, and we will only adopt this term in this manuscript for communication easiness [52].

2 Practitioners will consist of physicians, psychiatrists, and psychologists.

3 “PART-Beirat” stands for Participatory Beirat.

4 The time points refer to the time period after the establishment of the advisory boards and not the start of the research project.
